# Meeting Report: Hackathon-Workshop on Darwin Core and MIxS Standards Alignment (February 2012)

**DOI:** 10.4056/sigs.3166513

**Published:** 2012-09-28

**Authors:** Éamonn Ó Tuama, John Deck, Gabriel Dröge, Markus Döring, Dawn Field, Renzo Kottmann, Juncai Ma, Hiroshi Mori, Norman Morrison, Peter Sterk, Hideaki Sugawara, John Wieczorek, Linhuan Wu, Pelin Yilmaz

**Affiliations:** 1 Global Biodiversity Information Facility, GBIF Secretariat, Copenhagen, Denmark; 2 The University of California at Berkeley, Berkeley Natural History Museums, Berkeley, California, USA; 3 Botanic Garden & Botanical Museum Berlin-Dahlem, Freie Universität Berlin, Berlin, Germany; 4 Global Biodiversity Information Facility, GBIF Secretariat, Copenhagen, Denmark; 5 Molecular Evolution and Bioinformatics Group, Head Centre for Ecology & Hydrology, Wallingford, Oxfordshire, UK; 6 Microbial Genomics Group, Max Planck Institute for Marine Microbiology & Jacobs University Bremen, Bremen, Germany; 7 Institute of Microbiology, Chinese Academy of Sciences, Beijing, China; 8 Tokyo Institute of Technology, Department of Biological Information,Yokohama, Japan; 9 The University of Manchester, School of Computer Science, Oxford Road, Manchester, UK. M13 9PL; 10 NERC Environmental Bioinformatics Centre, Centre for Ecology & Hydrology, Maclean Building, Benson Lane, Crowmarsh Gifford, Wallingford, Oxfordshire, UK. OX10 8BB; 11 Oxford e-Research Centre, University of Oxford, Oxford, UK; 12 National Institute of Genetics, Mishima, Shizuoka, Japan; 13 Museum of Vertebrate Zoology University of California Berkeley, CA USA; 14 Institute of Microbiology, Chinese Academy of Sciences, Beijing, China; 15 Microbial Genomics Group, Max Planck Institute for Marine Microbiology & Jacobs University Bremen, Bremen, Germany

## Abstract

The Global Biodiversity Information Facility and the Genomic Standards Consortium convened a joint workshop at the University of Oxford, 27-29 February 2012, with a small group of experts from Europe, USA, China and Japan, to continue the alignment of the Darwin Core with the MIxS and related genomics standards. Several reference mappings were produced as well as test expressions of MIxS in RDF. The use and management of controlled vocabulary terms was considered in relation to both GBIF and the GSC, and tools for working with terms were reviewed. Extensions for publishing genomic biodiversity data to the GBIF network via a Darwin Core Archive were prototyped and work begun on preparing translations of the Darwin Core to Japanese and Chinese. Five genomic repositories were identified for engagement to begin the process of testing the publishing of genomic data to the GBIF network commencing with the SILVA rRNA database.

## Background

The Global Biodiversity Information Facility [1] (GBIF) Strategic Plan 2012-2016 [2] highlights the need to address the coming challenge and opportunity of making accessible information regarding the estimated 90% of the planet's biodiversity that is still to be discovered and shared, the currency of which will primarily be genomic biodiversity data. To this end, GBIF is collaborating with the Genomic Standards Consortium [3] (GSC) Biodiversity Working Group (GBWG) on common issues, principally around the alignment of standards. During February 27-29, 2012, GBIF led a joint hackathon-workshop on species-level biodiversity and genomic data standards with the aim of ensuring alignment and harmonization of efforts in these related domains and contributing to the ongoing work and series of workshops of the USA National Science Foundation funded Research Coordination Network (RCN) project for the GSC (RCN4GSC) [4] which seeks to promote the integration of genomic standards with ecological and species level standards. Hosted at the Oxford e-Research Centre [5], the workshop brought together a small group of experts from Europe, USA, China and Japan.

## Purposes of the Meeting

The goals of the workshop were to continue the process of aligning the Darwin Core [6] with the MIxS [7] and related genomic standards (e.g. ABCDDNA [8] and WFCC [9]), advance issues on vocabulary/ontology management including multilingual aspects, develop a DwC-A extension for serving genomic data, and identify suitable genomic data repositories with which to engage on connecting to the GBIF network.

### Participants

The participants were chosen for their technical knowledge of the various standards and genomic databases, although, in the case of the latter, it was not the intention to have representation of all the major repositories (see appended list).

### Outputs

**Vocabulary Alignment (DwC, MIxS, ABCDDNA, WDCM)**
1. The alignment (mapping) of the DwC and GSC MIxS checklists which had begun in previous workshops was completed (relevant terms from ABCDDNA and the WDCM were also considered), and an RDF expression of the MIxS terms was prepared. To express the application specific constraints in RDF, it will be necessary to apply practices set forth in “Expressing Dublin Core metadata using the Resource Description Framework (RDF)” http://dublincore.org/documents/dc-rdf/. The following outputs are available:
2. First draft of MIxS checklist (version 2011-01-26) in RDF: https://gist.github.com/1923079
3. First draft of MIxS checklist (version 2012-02-29) in RDF with MIxS term deprecations in favor of DwC terms: https://gist.github.com/1940237
4. MIxS, DwC, WDCM, ABCDDNA Mappings captured in Google Spreadsheet: http://goo.gl/esjYf
5. MIxS Quick Reference to terms including DwC terms: http://goo.gl/esjYf
6. MIxS with DwC term replacements Quick Reference spreadsheet (useful for doing source field to standard mappings) also in Mappings Document: http://goo.gl/esjYf
7. New download created on Darwin Core Code Site for a CSV file as a template for DwC term translations: http://code.google.com/p/darwincore/downloads/detail?name=DwCTermsForTranslations_2011-10-16.csv

### Vocabulary and Ontology Management

The use and management of controlled vocabulary terms was considered in relation to both GBIF and the GSC, and tools for working with terms were reviewed.

The MIxS standard [10] is maintained in a relational database system at the Max Planck Institute for Marine Microbiology Bremen on behalf of the GSC. This resource is not open for public access, but can be downloaded and installed locally - instructions in this document [7]). Further developments, extensions and enhancements for MIxS can be requested at a public issue tracking system at The Genomic Contextual Data Markup Language (GCDML) webpage [11]. Direct export to excel and GCDML is available, with RDF being another proposed format.

**Tools under initial review for working with vocabulary terms, included the following:**
- ISA Creator, tools to assign terms from ontologies and consume spreadsheet data
- Rightfield (http://www.sysmo-db.org/rightfield) propose terms from Ontologies and map to spreadsheet data. Clean interface, could not quite get it to work, need help.
- Terminizer (http://terminizer.org/) -- propose terms from Ontologies proof of concept
- Ontology Annotator (http://bioportal.bioontology.org/annotator)
- OntoFinder (http://ontofinder.dbcls.jp/)

It was discussed that a DwC-A exporter could be developed for one or more of these tools.

### Vocabulary Translation

A complete, authoritative list of current DwC terms needed for mapping data (thus without abstract terms, Class terms, or Type Vocabulary terms) was made available as a CSV file (http://code.google.com/p/darwincore/downloads/detail?name=DwCTermsForTranslations_2011-10-16.csv). This file is recommended as a starting point for translations or other further documentation for Darwin Core. The workshop offered opportunities for face to face discussions concerning translation issues. The teams addressing the translations to Japanese and Chinese completed their work after the workshop and provided the translation files to GBIF for merging into a SKOS document and publication on the GBIF vocabularies site. A draft SKOS document is available on the GBIF community site: http://community.gbif.org/pg/file/read/23196/darwin-core-translations-skos.

### DwC-A for genomic data

During the workshop, two extensions for publishing genomic biodiversity data to the GBIF network via a DwC-A were prototyped. Both of these use a DwC "occurrence" as the core data type. The extensions are “MIxS Sample” and “TaxonAbundance”.

**MIxS Sample:**
http://rs.gbif.org/sandbox/extension/mixs_sample.xml

**TaxonAbundance:**
http://rs.gbif.org/sandbox/extension/abundance.xml

Taxon assignment against metagenome sequences is indispensable for figuring out the entire behavior of the microbiome. Metagenome data are usually summarized as an abundance of each taxon in a sample using taxonomic assignment results of metagenome sequences from the sample [12]. The "TaxonAbundance" extension was developed to describe this taxonomic summary information of the sample via the DwC-A.

The workshop participants discussed measurements and facts that could be expressed within the scope of the existing “MeasurementOrFact” extension (http://rs.tdwg.org/dwc/terms/MeasurementOrFact). The Phenotypic Quality Ontology (PATO), Chemical Entities of Biological Interest (ChEBI), and Environment Ontology (EnvO) were discussed as ontologies providing a basis for the type of data while the Unit Ontology (UO) provides a means for expressing units within this extension [13].

Together, the suite of extensions above forms what we termed the “MIxS Profile”. The discussions have formed a starting point for further discussion in developing the MIxS Profile and further discussions have already ensued at the iDigBIO Workshop in Florida in March, 2012 regarding the composition of extensions in the profile and the properties defined within each extension.

## Genomic repositories

**Five genomic repositories were identified for engagement to begin the process of testing the publishing of genomic data to the GBIF network:**
1. WFCC (World Federation of Culture Collections); already a participant in GBIF (new MoU signed) but database has moved from Japan to China and there is requirement to work with Dr Juncai Ma to connect the new server.
2. SILVA [14] (http://www.arb-silva.de/) provides up to date, quality controlled databases of aligned rRNA sequences from the Bacteria, Archaea and Eukarya domains. All sequences have associated contextual information, multiple taxonomic classifications, and the latest validly described nomenclature.
3. MG-RAST [15] (http://metagenomics.anl.gov/), metagenomics Rapid Annotation using Subsystem Technology enables taxonomic and functional classification of metagenomic sequences.
4. Moorea Biocode Project (http://www.mooreabiocode.org/) is creating the first comprehensive inventory of all non-microbial life in a complex tropical ecosystem including construction of a library of genetic markers and physical identifiers for every species of plant, animal and fungi.
5. The MicrobeDB.jp project including MEO (http://mdb.bio.titech.ac.jp/meo/about_meo) coordinates microbial genomic and metagenomic data, and is supported by NBDC (National Bioscience Database Center, http://biosciencedbc.jp/nbdc.cgi?lng=en), Japan.

As a representative of SILVA was participating in the meeting (PY), it was possible to explore in some detail the structure of this database and its mapping to the DwC-A format, and in the weeks immediately following the workshop, a prototype export was completed and is currently being processed by GBIF. SILVA data was represented as a Darwin Core Occurrence, plus two extensions; Literature References and Identification History. As the core requires a taxonomic designation for each sequence, the SILVA classification was chosen. Alternative taxonomic opinions are represented in the Identification History extension. The core contains relevant rRNA sequence metadata parsed by SILVA from EMBL-ENA, which are mapped to relevant Darwin Core properties. For example, “collection_date” field is represented by verbatimEventDate, while “country” corresponds to locality. The Literature References extension contains the publication title, identifier, journal, as well as author information, if these were present alongside sequence records. Finally, the Identification History extension was used to represent the different taxonomic opinions for the sequences, i.e., the SILVA classification, and the Ribosomal Database Project II (RDP-II) classification.

Thanks to the development of the new/next generation sequencers, the number of sequences of microbial genes and genomes has literally exploded in recent years. In the meantime, pipelines for the annotation of sequences have been developed and served via the Internet to relieve the bottleneck in data mining of sequences, e.g. IMG [16], RAST [17], MiGAP [18]). Our next step, as a community, is to approach the developers of these pipelines to ensure conformance to the standards. This will greatly improve the quality and interoperability of diverse databases and contribute to the efficient re-use of data.

## Conclusions / Outcomes

A GBIF community site has been established to act as focal point for the group to continue collaborations: http://community.gbif.org/pg/groups/22216/genomic-biodiversity-data/. Membership is open to all (requires login) and all workshop participants have received an invitation to join. Several follow-on action items were identified and are being dealt with by the parties listed.

**The following tasks have been identified as the next steps in building on the outcomes of the workshop:**
1. ABCDDNA, MIxS, DwC: continue to investigate mapping/crosswalk (possibly via the Global Genome Biodiversity Network).
2. Create script to generate core RDF from GCDML database; publish RDF view of MIxS core (MIxSCore.rdf) on GSC site.
3. Explore option of Global Genome Biodiversity Network as forum for advancing biodiversity genomics in its broadest sense (not just tissue/biobanks/repositories).
4. With prototype DwC extensions now in place (as output of workshop) work with a few genomic databases/repositories to enable them to serve data to GBIF network. As first cases, it was decided (after review/discussion in workshop) to go with three initiatives: SILVA, MG-RAST and Moorea Biocode and expand out from there to include others. Initiate formal contacts with SILVA, MG-RAST and Moorea Biocode.
5. Re-connect the WFCC database, now moved from Japan to China, to GBIF network. Now that the WDCM is developing the WFCC Global Catalogue of Microorganisms (GCM), much more data from WFCC culture collections will be available to GBIF.
6. Deliver Japanese translation of DwC properties to GBIF.
7. Deliver Chinese translation of DwC properties to GBIF.
8. Publish SKOS version of DwC translations on GBIF site.
9. Prepare inputs to Semantics of Biodiversity workshop (Kansas).
10. Address vocabulary terms needing clarification.
11. Plan for RDF session at GSC14.
12. Describe encoding of constraints in an RDF document.
13. Prepare MIxS Profile guide.
